# Diphtheria in the Postepidemic Period, Europe, 2000–2009

**DOI:** 10.3201/eid1802.110987

**Published:** 2012-02

**Authors:** Karen S. Wagner, Joanne M. White, Irina Lucenko, David Mercer, Natasha S. Crowcroft, Shona Neal, Androulla Efstratiou, on behalf of the Diphtheria Surveillance Network

**Affiliations:** Health Protection Agency, London, UK (K.S. Wagner, J.M. White, N.S. Crowcroft, S. Neal, A. Efstratiou);; State Agency Infectology Center of Latvia, Riga, Latvia (I. Lucenko);; World Health Organization Regional Office for Europe, Copenhagen, Denmark (D. Mercer);; Public Health Ontario, Toronto, Ontario, Canada (N.S. Crowcroft);; University of Toronto Dalla Lana School of Public Health, Toronto (N.S. Crowcroft)

**Keywords:** diphtheria, Corynebacterium diphtheriae, Corynebacterium ulcerans, bacteria, toxin, surveillance, immunization, vaccination, pseudomembrane, antitoxin, epidemiology, Europe

## Abstract

Diphtheria incidence has decreased in Europe since its resurgence in the 1990s, but circulation continues in some countries in eastern Europe, and sporadic cases have been reported elsewhere. Surveillance data from Diphtheria Surveillance Network countries and the World Health Organization European Region for 2000–2009 were analyzed. Latvia reported the highest annual incidence in Europe each year, but the Russian Federation and Ukraine accounted for 83% of all cases. Over the past 10 years, diphtheria incidence has decreased by >95% across the region. Although most deaths occurred in disease-endemic countries, case-fatality rates were highest in countries to which diphtheria is not endemic, where unfamiliarity can lead to delays in diagnosis and treatment. In western Europe, toxigenic *Corynebacterium ulcerans* has increasingly been identified as the etiologic agent. Reduction in diphtheria incidence over the past 10 years is encouraging, but maintaining high vaccination coverage is essential to prevent indigenous *C. ulcerans* and reemergence of *C. diphtheriae* infections.

## Abstract 2

Efforts must be made to maintain high vaccination coverage.

In 1994, following success of widespread vaccination programs earlier in the century, diphtheria was proposed as a candidate for elimination in the World Health Organization (WHO) European Region; the goal was for elimination of indigenous diphtheria by 2000 (*1*). However, during the 1990s, when this goal seemed within sight, several factors caused a resurgence of diphtheria to epidemic proportions in the newly independent states of the former Soviet Union. There were a large number of unnecessary contraindications to vaccination in guidance for these countries at that time, which led to reductions in adequate vaccination coverage in children. This problem was exacerbated by mistrust in vaccinations among health professionals and the public and by use of low-dose formulation vaccine for primary vaccinations. Waning immunity in the adult population, large-scale population movements caused by breakup of the former Soviet Union, disruptions in health services, and lack of adequate supplies of vaccine and antitoxin for prevention and treatment in most affected countries provided conditions under which diphtheria could spread (*2**,**3*). At the peak of the epidemic in 1995, there were >50,000 cases reported in the WHO European Region (*2*). Intensive vaccination strategies brought the disease under control in most countries, but some endemic transmission still continues.

Clinical diphtheria is caused by toxin-producing corynebacteria. Three species (*Corynebacterium diphtheriae*, *C. ulcerans*, and *C. pseudotuberculosis*) can potentially produce diphtheria toxin. *C*. *diphtheriae* is the most common of potentially toxigenic species and is associated with epidemic diphtheria and person-to-person spread. The organism has 4 biovars (gravis, mitis, intermedius, and belfanti). *C*. *ulcerans* is historically associated with cattle or raw dairy products, and, although it is rarely reported, its incidence has increased slightly in some countries in western Europe and in the United States in recent years (*4**–**6*). *C*. *pseudotuberculosis* rarely infects humans and is typically associated with farm animals (*7*). Currently, no direct evidence has been found of person-to-person spread of *C*. *ulcerans* or *C*. *pseudotuberculosis*.

Classical respiratory diphtheria is characterized by formation of a gray-white pseudomembrane in the throat that is firmly adherent (*8*). A swollen, bull-neck appearance caused by inflammation and edema of soft tissues surrounding lymph nodes is associated with severe illness and higher death rates (*8*). In progressive disease, the toxin can bind to cardiac and nerve receptors and cause systemic complications. Milder respiratory disease may manifest as a sore throat, most commonly seen in patients who are fully or partially vaccinated. In some tropical areas, cutaneous symptoms, characterized by rolled-edge ulcers, are more common. Patients may have both cutaneous and respiratory disease. The purpose of this study was to analyze diphtheria data for Europe during 2000–2009.

## Methods

Case-based diphtheria surveillance data from each of 25 Diphtheria Surveillance Network (DIPNET) member countries (Austria, Belgium, Bulgaria, Cyprus, Czech Republic, Denmark, Estonia, Finland, France, Germany, Greece, Ireland, Italy, Latvia, Lithuania, the Netherlands, Norway, Poland, Portugal, Romania, Slovenia, Spain, Sweden, Turkey, and the United Kingdom) for 2000–2007 were submitted retrospectively to the coordinating center in the United Kingdom during 2008. Data for 2008 and 2009 were obtained in August 2009 and September 2010 from the DIPNET online database, which was launched in September 2007.

We analyzed cases meeting the DIPNET case definition (isolation of a toxigenic strain or clinically compatible case with an epidemiologic link to a laboratory-confirmed case) (Technical Appendix 2). In addition, 48 cases without laboratory confirmation and pseudomembrane (mild diphtheria/severe pharyngitis) and 5 cases with unknown manifestations were included for Latvia because these cases had been recorded in the national dataset. For most cases, toxigenicity was confirmed by using the Elek phenotypic test (*9*). However, in some cases, toxigenicity was evaluated only by detection of the toxin gene with PCR. We assumed that all cases in this dataset were toxigenic (toxin producing) because the number of cases without Elek confirmation was small and referred to symptomatic cases. Data fields collected included year; organism; biovar; and patient age, sex, clinical manifestations, vaccination status, veterinary contact, risk group, and outcome. Further strain characterization (ribotyping) was available for a limited number of isolates as part of a screening study in 10 DIPNET countries (*10*).

Cases were assigned to 5 clinical manifestation groups. These groups were classic respiratory diphtheria with pseudomembrane (the most serious form of the disease); mild diphtheria/severe pharyngitis (respiratory symptoms without the pseudomembrane); cutaneous (toxigenic organism isolated from skin lesions); other (e.g., toxigenic organism isolated from blood); and asymptomatic (carriers of toxigenic organisms, usually contacts of a confirmed case-patient).

**Figure 1:**
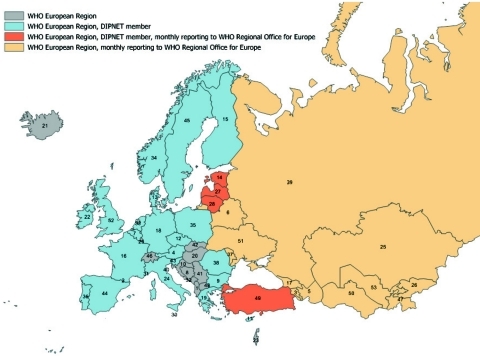
Diphtheria Surveillance Network (DIPNET) and World Health Organization (WHO) European Region countries. 1, Albania; 2, Andorra; 3, Armenia; 4, Austria; 5, Azerbaijan; 6, Belarus; 7, Belgium; 8, Bosnia and Herzegovina; 9, Bulgaria; 10, Croatia; 11, Cyprus; 12, Czech Republic; 13, Denmark; 14, Estonia; 15, Finland; 16, France; 17, Georgia; 18, Germany; 19, Greece; 20, Hungary; 21, Iceland; 22, Ireland; 23, Israel (neighboring countries not shown); 24, Italy, 25; Kazakhstan; 26, Kyrgyzstan; 27, Latvia; 28, Lithuania; 29, Luxembourg; 30, Malta; 31, Monaco; 32, Montenegro; 33, the Netherlands; 34, Norway; 35, Poland; 36, Portugal; 37, Republic of Moldova; 38, Romania; 39, Russian Federation; 40, San Marino; 41, Serbia; 42, Slovakia; 43, Slovenia; 44, Spain; 45, Sweden; 46, Switzerland; 47, Tajikistan; 48, Former Yugoslav Republic of Macedonia; 49, Turkey; 50, Turkmenistan; 51, Ukraine; 52, United Kingdom (Great Britain and Northern Ireland); 53, Uzbekistan.

Additional information concerning countries in the WHO European Region that are not DIPNET member countries was provided by the WHO Regional Office for Europe. Twenty-five of 53 member states of the WHO European Region are members of DIPNET. WHO European Region countries (including DIPNET members) report total cases annually to the WHO Regional Office for Europe through the WHO/United Nations Children’s Fund Joint Reporting Form, which is the global annual data survey of WHO member states for vaccine-preventable diseases and immunization program indicators. In addition, 16 countries in 2003 (Figure 1) were asked to prospectively participate in monthly surveillance and provide more detailed information (e.g., pathogen biovar; patient age, sex, and outcome; and carriers among contacts). Twelve countries currently provide monthly reports to WHO Regional Office for Europe through this system. The only major source of cases that has not participated in the monthly reporting system (but does report annually) is the Russian Federation. Rates per 1 million person-years were calculated by using population estimates derived from the Population Division of Economic and Social Affairs of the United Nations Secretariat (*11*).

### Statistical Analyses

Proportions were compared by using χ^2^ or Fisher exact tests, as appropriate, in Stata statistical software version 7.0 (StataCorp LP, College Station, TX, USA). For assessment of a trend for variables in ordered groups (vaccinated, partially vaccinated, unvaccinated) and severity of disease (classic respiratory, mild diphtheria/severe pharyngitis, asymptomatic), the Wilcoxon test for trend in Stata (*12*) was used. This test enabled nonparametric analysis across these groups.

## Results

**Figure 2:**
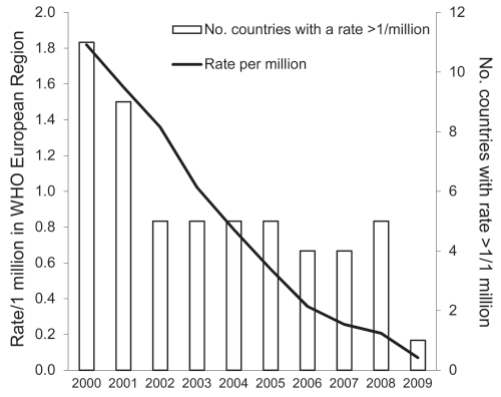
Diphtheria cases per 1 million population in the World Health Organization (WHO) European Region and number of countries with a rate >1 cases/1 million population, 2000–2009.

Overall, across the WHO European Region, the number of cases of diphtheria has substantially decreased since the epidemic in the 1990s (Figure 2). Data on clinically confirmed cases and toxigenic isolates of *C*. *diphtheriae* and *C*. *ulcerans* reported to DIPNET during 2000–2009 are shown in Table 1 and Table 2, respectively. Member countries that are not listed reported no isolates. Data are analyzed separately for Latvia, where diphtheria is endemic.

**Table 1:** Toxigenic *Cornyebacterium diphtheriae* isolates and epidemiologically linked cases and deaths reported by DIPNET member countries, Europe, 2000–2009*

Characteristic	Patient description†	No. toxigenic isolates or clinical cases with epidemiologic link (no. deaths)									
		2000	2001	2002	2003	2004	2005	2006	2007	2008	2009
Country											
Estonia	Symptomatic	2	2	0	0	0	0	0	0	0	0
	Asymptomatic	1	7	0	0	0	0	0	0	0	0
	Total	3	9	0	0	0	0	0	0	0	0
Finland	Total	0	2 (1)	0	0	0	0	0	0	0	0
France	Total	0	0	1	0	1	0	1	1 (1)	1	0
Germany	Total	1	2	4	0	0	1	0	0	0	2
Latvia	Symptomatic	145	0	45	26	20	20	32	18	29	6
	Asymptomatic	61	24	15	22	2	2	11	5	12	3
	Not known	119	91	0	0	0	0	0	0	0	0
	Total	325 (9)	115 (5)	60 (3)	48 (2)	22 (1)	22 (2)	43 (6)	23 (1)	41 (2)	9 (1)
Lithuania	Symptomatic	2	0	4	0	0	0	0	0	2	0
	Asymptomatic	0	0	1	0	0	0	0	0	2	0
	Total	2	0	5 (1)	0	0	0	0	0	4 (1)	0
Norway	Symptomatic	0	0	0	0	0	0	0	0	3	0
	Asymptomatic	0	0	0	0	0	0	0	0	1	0
	Total	0	0	0	0	0	0	0	0	4	0
Sweden	Total	0	0	0	0	0	0	0	0	0	1
Turkey	Symptomatic	1	3	1	0	0	0	0	0	0	0
	Asymptomatic	2	0	0	0	0	0	0	0	0	0
	Not known	1	4	1	1	0	0	0	0	0	0
	Total	4 (1)	7 (3)	2 (1)	1	0	0	0	0	0	0
United Kingdom	Total	1	0	6	3	0	0	1	0	2 (1)	2
Total known symptomatic patients	NA	152	9	61	29	21	21	34	19	37	11
Total (all countries)	NA	336 (10)	135 (9)	78 (5)	52 (2)	23 (1)	23 (2)	45 (6)	24 (2)	52 (4)	14 (1)
Total known symptomatic patients, excluding Latvia	NA	7	9	16	3	1	1	2	1	8	5
Total, excluding Latvia	NA	11 (1)	20 (4)	18 (2)	4	1	1	2	1 (1)	11 (2)	5

*DIPNET, Diphtheria Surveillance Network; NA, not applicable. A total of 89 cases were clinically diagnosed without microbiological confirmation (76 in Latvia, 11 in Turkey, and 2 in Lithuania). †If only total is displayed for a country, all patients were symptomatic.

**Table 2:** Isolates of toxigenic *Corynebacterium ulcerans* and patient deaths reported by DIPNET member countries, Europe, 2000–2009*

Characteristic	Patient description†	No. toxigenic isolates (no. deaths)									
		2000	2001	2002	2003	2004	2005	2006	2007	2008	2009
Country											
France	Total	0	1	0	1	3	0	2	1	0	1
Germany	Total	1	1 (1)	0	0	1	2	1	2	0	2
Italy	Total	0	0	1	0	0	0	0	0	0	0
The Netherlands	Total	0	1	0	0	0	0	0	1	0	0
Romania	Asymptomatic	0	0	1	0	0	0	0	0	0	0
	Total	0	0	1	0	0	0	0	0	0	0
Sweden	Symptomatic	0	0	0	0	0	0	0	0	1	0
	Not known	0	0	0	0	1	0	1	0	0	0
	Total	0	0	0	0	1	0	1	0	1	0
United Kingdom	Total	7 (1)	3	2	2	1	2	2 (1)	3	3	2
No. symptomatic patients	NA	8	6	3	3	5	4	5	7	4	5
No. isolates	NA	8 (1)	6 (1)	4	3	6	4	6 (1)	7	4	5

*DIPNET, Diphtheria Surveillance Network; NA, not applicable †If only total is shown for a country, all patients were symptomatic.

### Diphtheria-Endemic Countries in WHO European Region

During 2000–2009, Latvia reported the highest annual incidence rate of diphtheria in the European Region each year and a 10-year incidence rate of 23.8 cases/1 million person-years. This rate was ≈7× higher than in countries with the next highest 10-year incidence: i.e., Georgia (3.5), Ukraine (3.3), and the Russian Federation (3.0). However, during this time, 4,304 (>61%) of 7,032 cases in the WHO European Region were reported from the Russian Federation, and 2 countries, the Russian Federation and Ukraine, accounted for 83% of all cases.

Over the past 10 years, diphtheria incidence decreased by >95% across the region (from 1.82/1 million population in 2000 to 0.07/million in 2009), including in Latvia (from 111.22/million in 2000 to 2.67/million in 2009). In 2009, Latvia was the only country in the region that had not yet achieved the elimination benchmark of an incidence <1 case/million population (Figure 2).

Most cases reported to WHO through the monthly surveillance system were in teenagers and adults. However, the major risk groups for death have been infants (too young for complete primary vaccination) and adults >40 years of age (unvaccinated or with waning immunity). Although risk did not differ by sex in cases in children, during 2002–2009, ≈2× as many cases were reported in women >20 years of age than in men (510 [64%] vs. 292 [36%], respectively). Most (75%) case-patients reported in the European Region were at least partially vaccinated, but most (74%) case-patients and (93%) infants who died were unvaccinated). *C*. *diphtheriae* biovar gravis was the predominant strain (60%–80%). Of isolates from Latvia (Table 1), 355 (99%) of 358 with a known biovar were gravis and 3 (1%) were mitis.

Clinical manifestations and vaccination status for cases from Latvia (all *C*. *diphtheriae*) reported to DIPNET are shown in Table 3. Most (340/341) case-patients with symptoms had respiratory manifestations, and 141 (41%) of 340 respiratory case-patients had classic diphtheria symptoms. Vaccination showed a significant protective effect with respect to severity of infection (p<0.001 by test for trend).

**Table 3:** Vaccination status of case-patients and clinical manifestations of toxigenic *Corynebacterium diphtheriae* infections and epidemiologically linked cases without laboratory confirmation, Latvia, Europe, 2000–2009*

Vaccination status	Classic diphtheria (with membrane)	Mild diphtheria/ severe pharyngitis	Cutaneous	Asymptomatic	Not known	Total
Full	64†	118	0	71	0	253
Partial	1	3	0	5	0	9
Unvaccinated	74	70	1	18	0	163
Not known	2	8	0	63	210	283
Total	141	199	1	157	210	708

*p<0.001 by test for trend (vaccination status and disease severity). †Includes 52 fully vaccinated case-patients with classic respiratory diphtheria (with membrane) from an outbreak in the military in 2000. The outbreak comprised 145 symptomatic case-patients and 25 asymptomatic contacts. A total of 96% of these case-patients and contacts were 18–23 years of age at the time of diagnosis. Spread of disease was traced to use of a communal drinking cup (*13*).

**Figure 3:**
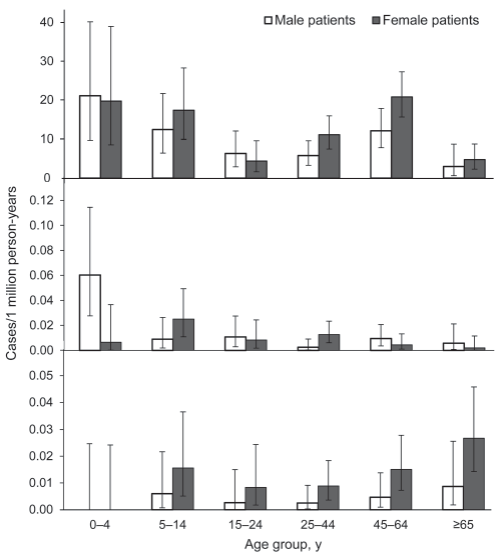
Diphtheria incidence per 1 million person-years for Latvia (*Corynebacterium diphtheriae*, 2002–2009) and the remaining 24 Diphtheria Surveillance Network (DIPNET) countries (*C. diphtheriae* and C*. ulceran*s, 2000–2009). Error bars indicate 95% CIs. The period 2002–2009 excludes the military outbreak in 2000 and cases from 2001 for which limited information was available.

For symptomatic cases for 2002–2009 (excluding the military outbreak in 2000 and cases from 2001 for which limited information was available) the highest overall incidences were in children 0–4 and 5–15 years of age and adults 45–64 years of age; lower incidence rates were observed in other age groups (Figure 3). Most (123/196, 63%) symptomatic cases during those years were in female patients.

The second most common risk factor (after military service) identified among symptomatic case-patients in Latvia was unemployment (60 case-patients). Information was not available regarding connections of case-patients to other countries of the former Soviet Union.

### Non–Disease-Endemic Countries (DIPNET)

Clinical manifestations and immunization status for case-patients with toxigenic *C*. *diphtheriae* and *C*. *ulcerans* isolates and epidemiologically linked cases reported by 24 DIPNET member countries, excluding Latvia, during 2000–2009 are shown in Table 4. Vaccination had a significant protective effect with respect to severity of infection (p = 0.001 by test for trend).

**Table 4:** Vaccination status of case-patients and clinical manifestations of toxigenic *Corynebacterium diphtheriae* and *C. ulcerans* infections and epidemiologically linked cases without laboratory confirmation, DIPNET cases excluding Latvia, Europe, 2000–2009*

Vaccination status	Classic respiratory diphtheria (with membrane)	Mild respiratory diphtheria/severe pharyngitis	Cutaneous	Other	Asymptomatic	Not known	Total
Full	4	17	2	1†	2	0	26
Partial	5	3	7	0	0	0	15
Unvaccinated	14	3	4	0	1	0	22
Not known	15	10	15‡	1§	12	11¶	64
Total	38	33	28	2	15	11	127

*DIPNET, Diphtheria Surveillance Network. p = 0.001 by test for trend (vaccination status and disease severity). †Bacterial endocarditis (*C. diphtheriae*, fully vaccinated) ‡One cutaneous case-patient also had a sore throat. §Isolation from blood (*C. ulcerans*, vaccination status not known). ¶Includes 2 case-patients infected with *C. diphtheriae* who died and are assumed to have respiratory symptoms without specific details available.

#### *C. diphtheriae* Isolates

Isolates of *C*. *diphtheriae* were sporadically reported in the 24 DIPNET member countries, excluding Latvia. Each year, 0–6 symptomatic cases of toxigenic *C*. *diphtheriae* infection were reported by each country (53 cases during 2000–2009). For each case-patient, 0–4 asymptomatic contacts were reported (14 in the 10-year period). Of 60 isolates with a biovar recorded during 2000–2009, a total of 32 were gravis and 28 were mitis. Seventeen cutaneous cases, 35 respiratory (24 classic respiratory) cases, and 1 case with other manifestations were reported. Most (15/17, 88%) cutaneous cases were caused by biovar mitis, and most (17/28, 61%) respiratory cases with a known biovar were caused by biovar gravis. Sixteen of 17 patients with cutaneous disease had recently returned from traveling, had contact with travelers, or were recent immigrants from a disease-endemic area, as was the situation for 12 of 35 patients with respiratory disease. One case-patient with bacterial endocarditis had contact with a relative who had recently traveled to Pakistan. For case-patients with *C*. *diphtheriae* symptomatic infection, sex distribution was even. A higher incidence rate was observed in male patients 0–4 years of age (Figure 3), but this finding was influenced by 6 cases reported in Turkey during 2001–2003.

#### *C. ulcerans* Isolates

A total of 4–8 isolations of toxigenic *C*. *ulcerans* were reported by DIPNET member countries each year (53 [50 symptomatic] during 2000–2009). Of these cases, 51% were reported by the United Kingdom, 19% by Germany, and 17% by France. Of the symptomatic cases for which patient sex/age group were known, 38 (78%) of 49 were in female patients and 29 (59%) of 49 were in patients >45 years of age. Incidence rate was higher in female patients than in than male patients (0.014/1 million person-years vs. 0.004/1 million person-years). Eleven cutaneous cases, 38 respiratory (14 classic respiratory) cases, and 1 case with other manifestations were reported. Ninety-four percent of case-patients for which information was available had contact with domestic animals. Traditional risk factors such as consumption of raw milk products were not reported, and no patients had a recent history of travel. One of the 2 case-patients infected with *C*. *ulcerans* who died in the United Kingdom had an identical strain of *C. ulcerans* to that isolated from a dog with which the patient had been in contact (*14*). A similar finding was observed in France for a nontoxigenic case reported in 2003 (*5**,**15*). In 2007, identical strains were isolated from a patient infected with *C*. *ulcerans* and her pig in Germany (*16*).

#### *C. pseudotuberculosis* Isolates

Four case-patients with diphtheria caused by toxigenic *C*. *pseudotuberculosis* were reported: 1 in France in 2005 and 1 in 2008, 1 in Germany in 2004, and 1 in United Kingdom in 2008. Three of these patients had cutaneous manifestations (1 was unvaccinated, 2 had an unknown vaccination status) and 1 (partially vaccinated) had bacterial endocarditis. To our knowledge, none of these infected patients died. Animal contact (with a calf) was recorded for only 1 patient (1 had no history of animal contact and 2 had an unknown history of animal contact).

### Deaths Caused by Diphtheria

During 2000–2009, a total of 32 deaths caused by diphtheria were reported in Latvia, and 13 deaths (10 caused by *C*. *diphtheriae* and 3 caused by *C*. *ulcerans*) (Table 1, Table 2) were reported by the remaining 24 DIPNET countries. Overall, patients with respiratory disease and a pseudomembrane had a significantly higher case-fatality rate (CFR) than patients with respiratory disease without a pseudomembrane (14.6% vs. 1.3%; p<0.001). For case-patients in Latvia, the CFR was 5% for patients with any respiratory symptom (including classic manifestations) and 12% for patients with classic respiratory symptoms. Of 18 case-patients in Latvia who died, 14 were >40 years of age and 4 were <7 years of age; all were unvaccinated.

Nine of 13 patients who died of diphtheria in DIPNET countries excluding Latvia had classic respiratory diphtheria symptoms, and 2 had severe pharyngitis (2 had unknown manifestations). All 3 deaths caused by *C*. *ulcerans* (2 in the United Kingdom and 1 in Germany) were in elderly (>75 years of age) patients (unvaccinated or vaccination status unknown). Two of the patients infected with *C*. *diphtheriae* who died were unvaccinated infants (1 from Mayotte and 1 from Finland). The infant in Finland died at 3 months of age in 2001 after recent contact with visitors from Russia (*17*). Six other children died: an unvaccinated school age child in the United Kingdom (*18*) and 5 children <7 years of age in Turkey (vaccination status unknown). Two adults in Lithuania (ages 45–64 years; vaccination status unknown) also died. The CFR for patients with any respiratory symptoms reported for patients infected with toxigenic *C*. *diphtheriae* or *C*. *ulcerans* in regions where diphtheria was not endemic was 15%; CFR was 24% among patients with classic respiratory diphtheria.

The difference between CFRs for respiratory diphtheria cases in Latvia and member countries excluding Latvia (5% and 15%, respectively) was significant (p = 0.002). The difference between CFRs for classic respiratory diphtheria in Latvia and the member countries excluding Latvia (12% and 24%, respectively) showed borderline significance (p = 0.06).

Any case-patients without symptoms recorded who died likely had respiratory diphtheria. However, because symptoms were also not available for several surviving case-patients for whom clinical manifestations were less certain, all case-patients for whom clinical manifestations were unavailable were excluded from analysis.

## Discussion

Substantial progress has been made in controlling diphtheria across Europe since the epidemic in the 1990s, but diphtheria has not disappeared as a serious public health threat. After major disruption to a mass vaccination program, recovery time is lengthy, and pockets of unvaccinated persons can remain because recovery is not necessarily homogeneous.

The protective effect of vaccination in preventing progression to severe disease is clear. However, 64 patients in Latvia recorded as fully vaccinated had classic respiratory diphtheria symptoms. Most of these patients were infected during a military outbreak in 2000 and would have been scheduled for primary vaccinations during the 1980s, when changes in vaccines, vaccination policy, medical practice, and public acceptance led to less intensive vaccination of children in the former Soviet Union. Beginning in 1980, Soviet vaccination recommendations enabled use of an alternative primary vaccination schedule against diphtheria that recommended 3 doses of a lower-potency vaccine (*19*). The classification of fully/partially vaccinated relies on specific interpretation of a country. Since the 2000 outbreak, greater attention has been given to checking vaccination records of new recruits into the Latvian military, and booster vaccinations are given where appropriate.

Lower CFRs for respiratory diphtheria in disease-endemic areas compared with those in nonendemic areas highlight how lack of familiarity with a rare disease can affect diagnosis and treatment. As the incidence of diphtheria has decreased, so has the practice of routine laboratory screening (*20*). No DIPNET member country routinely screens all throat swab specimens for corynebacteria, although sentinel screening of all throat swab specimens is conducted in Denmark, Ireland, and the United Kingdom. All other DIPNET countries (and outside sentinel screening areas) perform screening only at the request of the clinician or if the laboratory identifies particular criteria for screening from information accompanying a swab specimen (DIPNET, unpub. data). This practice has resulted in a loss of laboratory expertise and the opportunity for infections to go undetected because only clinically indicated swab specimens are tested; thus, milder cases or those with unusual manifestations may be missed.

A recent DIPNET external quality assurance evaluation of 6 simulated throat specimens found that only 6 of 34 international centers produced acceptable results for all 6 specimens; many centers could not isolate the target organism (*21*). In some poor countries, screening can be limited by cost of laboratory reagents, and problems have also occurred in obtaining Elek reagents and media (*21*). During a recent screening study across 10 countries in Europe, toxigenic organisms were isolated in Latvia and Lithuania (*10*). At least one of these cases in Lithuania would not have been correctly diagnosed in the absence of the screening study. In addition to the potential for missed or late diagnoses, in areas where diphtheria is not endemic, diphtheria antitoxin treatment is not always available, which can have serious consequences. A recent international survey highlighted global shortages of diphtheria antitoxin (*22*). Information about administration and timing of antitoxin treatment was not collected for this analysis, but studying such timing in relation to differing CFRs would be useful.

Higher incidence rates of *C. diphtheriae* among women in disease-endemic countries could be caused by several factors. Women more commonly work as caregivers in domestic and health care settings, consultation rates are usually higher among women, and men are more likely to have received diphtheria vaccine during military service.

Although the United Kingdom, France, and Germany regularly report isolations of toxigenic *C. ulcerans*, it is unlikely that this organism is present only in these countries. The ability to detect *C. ulcerans* could indicate the capability of a country to detect potentially toxigenic organisms and provide an indicator of good surveillance. Detection of mild diphtheria cases (any toxigenic organism) is another potential indicator of good surveillance. *C*. *ulcerans* appears to have a wide host range and has been isolated from many domestic and wild animals, including the killer whale and lion (nontoxigenic strain) (*23*). During 2002 and 2003, toxigenic *C*. *ulcerans* strains isolated from domestic cats in the United Kingdom were found to have the predominant ribotypes observed among human clinical isolates, which suggests that cats could be a potential reservoir for human infection (*24*). Identical *C*. *ulcerans* strains have been isolated from diphtheria patients and dogs in France and the United Kingdom (*14**,**15*). The presence of this organism reinforces the need to maintain high vaccination levels in all countries. Higher incidence of infection among elderly women could be related to pet ownership habits, in combination with low or waning immunity.

Vaccination coverage for diphtheria is assessed annually in many countries in Europe by using a range of methods, including computerized vaccination registers, survey methods, administrative methods, or a combination (*25*). These methods will provide varying degrees of accuracy in coverage estimates, which makes countries difficult to compare. Coverage for vaccination with diphtheria-tetanus-pertussis 3 vaccine (third dose of diphtheria, tetanus, pertussis vaccine) in early childhood in 2009 was >90% for most (85%) countries in the European Region, and 66% of countries (including Latvia, Lithuania, Turkmenistan, and the Russian Federation) reported coverage >95% (*26*). Coverage in Ukraine decreased from 98% in 2006 and 2007 to 90% in 2008 and 2009. Austria, Denmark, Georgia, and Moldova recorded diphtheria-tetanus-pertussis 3 vaccine coverage <90%. Azerbaijan and Malta had the lowest coverage (73% for both countries) in the European Region in 2009.

Following high-profile vaccine-scare stories in some countries in eastern Europe, such as the Russian Federation and Ukraine, anti-vaccination groups have gained strength by using television, the Internet, and other media for publicity (*27*); this activity could seriously affect vaccination coverage. Adult diphtheria immunity can be increased through scheduled booster vaccinations every 10 years (e.g., as in Austria, Belgium, Bulgaria, Cyprus, Estonia, Finland, France, Germany, Greece, Latvia, Norway, Portugal, and Romania) or as part of a combined tetanus and low-dose diphtheria vaccine given for tetanus-prone injuries. In Latvia, annual adult vaccination coverage surveys are undertaken, but in most countries adult coverage is rarely assessed. Seroprevalence studies have indicated that many adults in some countries have immunity levels below the protective threshold (*28*). Gaps in immunity in the adult population contributed to the resurgence of diphtheria in eastern Europe during the 1990s.

Trends in diphtheria cases in Europe are encouraging, but continued striving for improved vaccination coverage is essential. Diphtheria has a socioeconomic component; outbreaks are typically seen in marginalized groups. In the current economic climate, more socially deprived groups that are vulnerable to infection will emerge. The economic crisis may also threaten supplies of vaccine and antitoxin and delivery of immunization programs. Because reductions in finances can limit the capacity for surveillance, decreases in case reporting need to be interpreted with caution. Every effort must be made to maintain high diphtheria vaccination coverage.

## Supplementary Material

Technical Appendix 1 Additional members of the Diphtheria Surveillance Network who contributed data.

Technical Appendix 2 European Union Case Definition for National Diphtheria Surveillance, Community Decision of March 19, 2002 (under 2119/98/EC), Modified Version.
