# Supplementary material for: Diphtheria in the Postepidemic Period, Europe, 2000–2009
Source: Emerg Infect Dis. 2012 Feb;18(2):217–25. doi: 10.3201/eid1802.110987 (PMC3310452; doi:10.3201/eid1802.110987)
Supplement: Technical Appendix 1 — Additional members of the Diphtheria Surveillance Network who contributed data. [file 11-0987-Techapp1_2p.pdf]

Article DOI: http://dx.doi.org/10.3201/eid1802.110987

Diphtheria in the Postepidemic Period, Europe, 2000–2009

Technical Appendix 1

Additional members of the Diphtheria Surveillance Network who contributed data: Ulrich Sagel (Instiut fur Medizinische Mikrobilogie und Hygiene, Linz, Austria); Martine Sabbe (Scientific Institute of Public Health, Brussels, Belgium); Ingrid Wybo (Universitaire Ziekenhuis Brussel–Vrije Universiteit, Brussels, Belgium); Antoaneta Decheva, Nadezhda Vladimirova (National Centre of Infectious and Parasitic Diseases, Sofia, Bulgaria); Chrystalla Hadjianastassiou (Ministry of Health, Nicosia, Cyprus); Despo Pieridou-Bagatzouni (Nicosia General Hospital, Nicosia, Cyprus); Bohumir Kriz (Charles University, Prague, Czech Republic and National Institute of Public Health, Prague, Czech Republic); Jana Zavadilova (National Institute of Public Health, Prague, Czech Republic); Peter Henrik Andersen (Statens Serum Institut, Copenhagen, Denmark); Jens Jørgen Christensen (Slagelse Hospital, Slagelse, Denmark); Unna Jõks and Irina Donchenko (Health Protection Inspectorate, Tallinn, Estonia); Markku Kuusi and Jaana Vuopio (National Institute for Health and Welfare, Helsinki, Finland); Nicole Guiso and Patrick Grimont (Institut Pasteur, Paris, France); Isabelle Bonmarin (Institut de Veille Sanitaire, Saint Maurice, France); Andreas Sing (National Consiliary Laboratory on Diphtheria, Bavarian Health and Food Safety Authority, Oberschleißheim, Germany); Wiebke Hellenbrand (Robert Koch Institute, Berlin, Germany); Jenny Kremastinou (National School of Public Health, Athens, Greece); Hellen Alexandrou-Athanasouli (Aghia Sophia Children's Hospital, Athens, Greece); Suzanne Cotter (Health Services Executive–Health Protection Surveillance, Dublin, Ireland); Philip Murphy (Adelaide and Meath National Children's Hospital, Dublin, Ireland); Cristina Rota and Christina von Hunolstein (Istituto Superiore di Sanità, Rome, Italy); Ruta Paberza and Jurijs Perevoscikovs (State Agency Infectology Center of Latvia, Riga, Latvia); Snieguole Dauksiene and Nerija Kuprevičiene (Centre for Communicable Diseases Prevention and Control, Vilnius, Lithuania); Hester de Melker and Frans Reubsaet (Centre for Infectious Disease Control, Bilthoven, the Netherlands); Ernst Arne Høiby and Per Sandven (Norwegian Institute of Public Health, Oslo, Norway); Aleksandra A. Zasada (National

Institute of Hygiene, Warsaw, Poland); Paula Lavado and M. João Simões (National Institute of Health Dr. Ricardo Jorge, Lisbon, Portugal); Aurora Stanescu (National Institute of Public Health, Bucharest, Romania); Cerasela Dragomirescu (National Institute of Research and Development for Microbiology and Immunology Cantacuzino, Bucharest, Romania), Alenka Kraigher and Verica Mioc (National Institute of Public Health, Ljubljana, Slovenia); Isabel Pena Rey (National Epidemiology Centre, Madrid, Spain); Birgitta Henriques Normark (Swedish Institute for Infectious Disease, Solna, Sweden); Selin Nar Otgun and Efsun Akbaş (Refik Saydam National Hygiene Centre, Ankara, Turkey); and Mehmet Ali Torunoglu (Turkish Ministry of Health, Ankara, Turkey)
