# Supplementary material for: Diphtheria in the Postepidemic Period, Europe, 2000–2009
Source: Emerg Infect Dis. 2012 Feb;18(2):217–25. doi: 10.3201/eid1802.110987 (PMC3310452; doi:10.3201/eid1802.110987)
Supplement: Technical Appendix 2 — European Union Case Definition for National Diphtheria Surveillance, Community Decision of March 19, 2002 (under 2119/98/EC), Modified Version. [file 11-0987-Techapp2_2p.pdf]

Article DOI: http://dx.doi.org/10.3201/eid1802.110987

# Diphtheria in the Postepidemic Period, Europe, 2000–2009

## Technical Appendix 2

European Union Case Definition for National Diphtheria Surveillance, Community Decision of March 19, 2002 (under 2119/98/EC), Modified Version.

Androulla Efstratiou, Natasha S. Crowcroft, and Joanne M. White, on behalf of Diphtheria Surveillance Network, November 2002

## Clinical Description

Clinical picture compatible with diphtheria, i.e., an upper respiratory tract illness characterized by sore throat, low-grade fever, and an adherent membrane of the tonsils, pharynx, or nose or nonrespiratory diphtheria; cutaneous, conjunctival, otic, and genital lesions.

## Laboratory Criteria for Diagnosis

Isolation of diphtheria toxin–producing corynebacteria from a clinical specimen.

## Case Classification

Possible: Not applicable

Probable: A clinically compatible case that is not laboratory confirmed and does not have an epidemiologic link to a laboratory-confirmed case.

Confirmed: A clinically compatible case that is laboratory confirmed with the isolation of a toxigenic strain of *Corynebacterium diphtheriae, C. ulcerans, or C. pseudotuberculosis* or has an epidemiologic link to a laboratory-confirmed case.

Confirmed (other): Nonrespiratory/cutaneous diphtheria cases with isolation of toxigenic strains, or cases not meeting the specified clinical criteria but with isolation of toxigenic strains (e.g., mild respiratory diphtheria, or respiratory diphtheria with absence of membrane).
Asymptomatic carriers: asymptomatic carriers (any anatomical site) with toxigenic strains.
